# Magnetic Fe@FeO_x_, Fe@C and α-Fe_2_O_3_ Single-Crystal Nanoblends Synthesized by Femtosecond Laser Ablation of Fe in Acetone

**DOI:** 10.3390/nano8080631

**Published:** 2018-08-20

**Authors:** Dongshi Zhang, Wonsuk Choi, Yugo Oshima, Ulf Wiedwald, Sung-Hak Cho, Hsiu-Pen Lin, Yaw Kuen Li, Yoshihiro Ito, Koji Sugioka

**Affiliations:** 1 RIKEN Center for Advanced Photonics, 2-1 Hirosawa, Wako, Saitama 351-0198, Japan; dongshi17@126.com (D.Z.); cws@kimm.re.kr (W.C.); 2 Department of Nano-Mechatronics, Korea University of Science and Technology (UST), 217 Gajeong-Ro, Yuseong-Gu, Daejeon 34113, Korea; shcho@kimm.re.kr; 3 Department of Nano-Manufacturing Technology, Korea Institute of Machinery and Material (KIMM), 156 Gajeongbuk-Ro, Yuseong-Gu, Daejeon 34103, Korea; 4 Condensed Molecular Materials Laboratory, RIKEN Cluster for Pioneering Research, 2-1 Hirosawa, Wako, Saitama 351-0198, Japan; yugo@riken.jp; 5 Faculty of Physics and Center for Nanointegration Duisburg-Essen (CENIDE), University of Duisburg-Essen, 47057 Duisburg, Germany; ulf.wiedwald@uni-due.de; 6 Department of Laser & Electron Beam Application, Korea Institute of Machinery and Material (KIMM), 156 Gajeongbuk-Ro, Yuseong-Gu, Daejeon 34103, Korea; 7 Emergent Bioengineering Materials Research Team, RIKEN Center for Emergent Matter Science, 2-1 Hirosawa, Wako, Saitama 351-0198, Japan; hsiu-pen.lin@riken.jp (H-P.L.); y-ito@riken.jp (Y.I.); 8 Department of Applied Chemistry, National Chiao Tung University, Science Building 2, 1001 Ta Hsueh Road, Hsinchu 300, Taiwan; ykl@cc.nctu.edu.tw; 9 Nano Medical Engineering Laboratory, RIKEN Cluster for Pioneering Research, 2-1 Hirosawa, Wako, Saitama 351-0193, Japan

**Keywords:** hematite α-Fe_2_O_3_, core-shell, blocking temperature, superparamagnetism, laser ablation in liquids, femtosecond laser, single-crystalline

## Abstract

There are few reports on zero-field-cooled (ZFC) magnetization measurements for Fe@FeO_x_ or FeO_x_ particles synthesized by laser ablation in liquids (LAL) of Fe, and the minimum blocking temperature (T_B_) of 120 K reported so far is still much higher than those of their counterparts synthesized by chemical methods. In this work, the minimum blocking temperature was lowered to 52 K for 4–5 nm α-Fe_2_O_3_ particles synthesized by femtosecond laser ablation of Fe in acetone. The effective magnetic anisotropy energy density (K_eff_) is calculated to be 2.7–5.4 × 10^5^ J/m^3^, further extending the K_eff_ values for smaller hematite particles synthesized by different methods. Large amorphous-Fe@α-Fe_2_O_3_ and amorphous-Fe@C particles of 10–100 nm in diameter display a soft magnetic behavior with saturation magnetization (M_s_) and coercivities (H_c_) values of 72.5 emu/g and 160 Oe at 5 K and 61.9 emu/g and 70 Oe at 300 K, respectively, which mainly stem from the magnetism of amorphous Fe cores. Generally, the nanoparticles obtained by LAL are either amorphous or polycrystalline, seldom in a single-crystalline state. This work also demonstrates the possibility of synthesizing single-crystalline α-Fe_2_O_3_ hematite crystals of several nanometers with (104), (113), (116) or (214) crystallographic orientations, which were produced simultaneously with other products including carbon encapsulated amorphous Fe (a-Fe@C) and Fe@FeO_x_ core-shell particles by LAL in one step. Finally, the formation mechanisms for these nanomaterials are proposed and the key factors in series events of LAL are discussed.

## 1. Introduction

The newly emerged technique of laser ablation in liquids (LAL) [1,2,3,4,5] has proven to be valid for the synthesis of a large variety of colloids resulting from material removal of the substances [6]. With specific targets, simply changing the liquids for LAL allows the easy alteration of colloidal properties such as sizes [7,8] and phases [9,10,11]. For the synthesis of magnetic particles by LAL, the most frequently investigated material is Fe, whose results have turned out to be very diverse depending on experimental conditions [2], in which nanosecond (ns) lasers were typically used. For example, Vahabzadeh and Torkamany made use of ns lasers at fundamental and 2nd harmonic wavelengths (1064 nm and 532 nm) to ablate Fe in water for the formation of Fe_3_O_4_ and FeO particles, whose saturation magnetizations (M_s_) and coercivity (H_c_) were 22.5 emu/g and 11.5 Oe and 14.8 emu/g and 22 Oe [12], respectively. These values were much lower than those of bulk magnetite (M_s_ = 92 emu/g and H_c_ = 500–800 Oe). Zeng et al. prepared FeO nanoparticles by ns laser fragmentation in liquid (LFL) of Fe using a water/poly(vinyl pyrrolidone) (PVP) solution [13]. Amendola et al. obtained FeO_x_ (Fe_3_O_4_, FeO, α-Fe) nanoparticles with an M_s_ of 100 emu/g by ns LAL in water [14]. Pandey et al. found that the ns LAL of commercial Fe_2_O_3_ powders in doubly distilled water improved the hematite particle crystallinity and increased M_s_ from 0.024 to 3.41 emu/g [15]. Svetlichnyi et al. found that the H_c_ of the FeO_x_ (Fe, Fe_2_O_3_ and Fe_3_O_4_) nanoparticles synthesized by ns LAL of Fe in water increased from 144 Oe to 370 Oe when the measurement temperature was reduced from 300 K to 77 K [16]. Ismail et al. showed that the M_s_ values of 16.3–20.3 emu/g for the magnetic iron oxide (Fe_3_O_4_, α-Fe_2_O_3_, FeO and ε-Fe_2_O_3_) nanoparticles synthesized by ns LAL in SDS aqueous solution were larger than 13.8–16.2 emu/g for FeO_x_ (Fe_3_O_4_, α-Fe_2_O_3_, ε-Fe_2_O_3_) particles synthesized by ns LAL in dimethylformamide (DMF) at the same laser energies [17]. Meanwhile, Kanitz et al. employed a femtosecond (fs) laser for LAL of Fe and reported that the products changed depending on the adopted solutions: α-iron, wüstite and magnetite were synthesized in water, α-iron, cementite and FeO_x_ in methanol, amorphous-Fe and α-Fe mixture in ethanol and acetone, and amorphous-Fe@C core-shell particles in toluene [18]. Their M_s_ and H_c_ values were measured to be 23, 80, 60, 67 and 14 emu/g, and 77, 92, 65, 56, and 52 Oe at 300 K, respectively. Santillán et al. synthesized FeO_x_ (Fe_3_O_4_, γ-Fe_2_O_3_ or α-Fe) by fs laser (120 fs, 1 kHz, 800 nm) ablation of Fe in water, by which M_s_, number density, magnetic radius and total radius of 49.3 emu/g, 2.9 × 10^18^, 1.1 nm, 1.9 nm, respectively, were obtained, which were different from the magnetic properties (26.7 emu/g, 3468 μ_B_ and 0.7 × 10^18^, 1.5 nm, 3.2 nm) of the particles obtained by LAL in a trisodium citrate aqueous solution [19]. Most of these works mainly focused on the hysteresis curves to reveal the dependence of magnetic properties on the phases of LAL-prepared FeO_x_ or Fe/FeO_x_ particles. Little attention has been paid to the zero-field-cooled (ZFC) and field-cooled (FC) curves including the information about blocking temperature (T_B_) which is closely related to the product of particle size and magnetic anisotropy energy density essentially defining the energy barrier between two easy axes of magnetization [20].

Amendola et al. observed a blocking temperature T_B_ = 200 K of FeO_x_ particles synthesized by the ns-LAL of Fe in water [14]. Franzel et al. reported that Fe_3_O_4_ and Fe_3_C synthesized by picosecond (ps) LAL of Fe in ethanol had a blocking temperature T_B_ = 120 K [21], much lower that of FeO_x_ particles synthesized by ns-LAL, which was due to the generation of higher ratios of smaller particles by ps-LAL. As is well known, a low T_B_ value (<100 K) is a good indicator for superparamagnetism arising from small particles [22,23]. The endowment of superparamagnetism to the as-prepared magnetic particles often requires the particle size to be around or less than 10 nm [24]. To date, the minimum blocking temperature of LAL-generated Fe@FeO_x_ or FeO_x_ particles is still above 100 K [21]. Due to the fact that smaller FeO_x_ particles often possess lower blocking temperatures [20], the synthesis of ultrasmall Fe@FeO_x_ and FeO_x_ particles by LAL is essential to lower the blocking temperature below 100 K. To this end, fs-LAL is a better choice than ps- and ns-LAL because of the phase/Coloumb explosion mechanism for fs-LAL rather than the thermal ablation mechanism for ns-LAL [25].

Despite many reports on the fs-LAL of Fe and studies on the magnetic properties (M_s_, M_r_ and H_c_) of the products [11,18], the ZFC/FC curves were not measured. To fill this gap and to further lower the blocking temperature of Fe@FeO_x_ possessing superparamagnetic properties, the fs-LAL of Fe in acetone was performed. XRD, high-resolution transition electron microscropy (HRTEM), energy-dispersive X-ray (EDX), selected area electron diffraction (SAED), fast Fourier transform (FFT), X-ray photoelectron spectroscopy (XPS) and Raman characterizations were performed to clarify the composition of the as-prepared particles. TEM analysis was performed to display the particle morphologies and calculate the size distribution of the colloids. Both ZFC/FC and hysteresis curves of the synthesized magnetic particles were measured, from which T_B_, M_s_, M_r_ and H_c_ values were determined.

## 2. Materials and Methods

Colloids were synthesized by laser ablation of a Fe sheet (99.45 wt % Fe, 0.42 wt % O, 0.13 wt % C) using a fs laser system (FGPA *μ*Jewel D-1000-UG3, IMRA America Inc., Ann Arbor, MI, USA). The pulse duration, wavelength and repetition rate of the laser system were 457 fs, 1045 nm and 100 kHz, respectively. An Fe sheet with dimensions of 20 mm × 20 mm × 1 mm was placed inside a glass container and then immersed in 8 mL acetone for LAL. The liquid thickness above the target surface was kept at 5 mm. Then, a fs laser beam was focused on the Fe sheet surface by a 20× objective lens (numerical aperture (NA) = 0.4, Mitutoyo, Kawasaki, Japan) and scanned over an area of 3.5 × 3.5 mm^2^ using the scan method described in [26,27,28] with a line interval of 5 μm and a scan speed of 1 mm/s to ablate the Fe sheet. The ablation process lasted around 1 h. The average laser power was set to 600 mW. The spot size was 26 μm. The peak irradiance and laser fluence were calculated to be 1.13 × 10^9^ W/m^2^ and 113 J/cm^2^, respectively.

The colloids were directly deposited onto TEM grids (EMJapan, U1015, Tokyo, Japan, 20 nm thick carbon films on copper grids) after LAL without any pre-treatment and then characterized using TEM (Jeol, JEM-1230, Tokyo, Japan) operating at 80 kV. HRTEM and STEM-EELS (scanning transmission electron microscopy–electron energy loss spectroscopy) were performed with a JEM-ARM200F (Jeol, Tokyo, Japan) equipped with third-order aberration correctors for both illuminating and imaging lens systems operated at 200 kV. For XRD and magnetic property measurements, the colloids with liquids were centrifuged by a centrifuge (Eppendorf, Centrifuge 5430, Hamburg, Germany) at a rotation speed of 14,000 rpm for 10 min. The precipitated particles were then collected in a cuvette and dried in a freeze dryer (Rikakikai, S-1000, Eyela, Tokyo, Japan). The dried particles were deposited on an amorphous glass plate (10 mm × 10 mm × 1 mm) for XRD, XPS and Raman characterizations. The composition of the particles was analysed using XRD (Rigaku, CuKα radiation (40 kV-30 mA), SmartLab-R 3kW, Tokyo, Japan). The surface chemistry of the particles was analysed by XPS (Thermo Scientific, ESCALAB 250, Tokyo, Japan) and Raman spectroscopy (LabRAM, Hiriba, He-Ne laser, 632 nm, 0.686 mW, Tokyo, Japan). A zeta-potential and particle size analyzer (ELSZ-2PL, Photal, Osaka, Japan) was used to measure the zeta potential of the fresh colloid and the colloid stored after 3 weeks. UV-vis spectroscopy (Shimadzu, UV-3600 Plus, Tokyo, Japan) was used to measure the absorption spectra of colloids.

Magnetic properties of the particles were measured in He gas atmosphere using the superconducting quantum interference device (SQUID) magnetometer (Quantum Design, MPMS XL7, San Diego, USA). The dried 26.5 mg Fe@α-Fe_2_O_3_ particle powder was filled into a capsule, which was then placed into the magnetometer. Zero-field-cooled (ZFC) magnetization was measured by cooling samples in a zero magnetic field and then increasing the temperature from 5 K to 300 K with magnetization-temperature data recorded every 5 K at an applied field of 50 Oe. Field-cooled (FC) curves were recorded by cooling the samples from 300 K to 5 K with a constant field of 50 Oe. The field dependence of the magnetization (hysteresis loop) was recorded up to ±70 kOe at T = 5 K and ± 10 kOe at T = 300 K, respectively.

## 3. Results

### 3.1. Material Property

**Figure 1:**
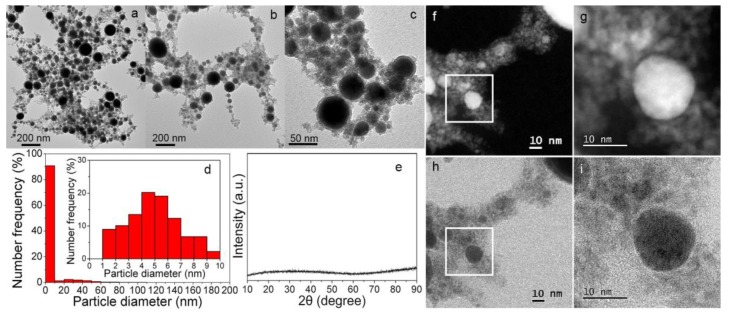
(**a**–**c**) TEM images of the particles synthesized by the laser ablation of Fe in acetone at 400 mW. (**d**) Size distribution of the synthesized particles. The inset figure shows the detailed size distribution in the range of 0–10 nm. (**e**) XRD pattern of the synthesized particles, where no peaks were detected, probably due to the low crystallinity of the particles and a large amount of carbon clusters. (**f**,**g**) and (**h**,**i**) black field and white field scanning transmission electron microscopy (STEM) images of small particles, respectively.

After drying on TEM grids, the particles synthesized in acetone form a particle network (Figure 1a–c), connected by a large amount of small clusters, which is a typical phenomenon after ferro-fluidic colloid drying [29,30,31,32,33,34]. The average size of the particles is estimated to be 5–6 nm (Figure 1d). Small particles with sizes of less than 10 nm occupy more than an 87% number frequency of all of the particles. In particular, small particles with a ~ 90% number frequency of 1–10 nm are in the majority, with the highest number frequency at 4–5 nm (Figure 1f–i). The large particles are in the form of core-shell particles with a shell thickness of ca. 6 nm (Figure 1c). No crystalline peak was observed in the XRD spectrum (Figure 1e), similar to the case of the nanomaterials obtained by LAL of Cu in acetone [35]. This is either due to the low amount of particle powders used for the XRD spectrum or due to the too-small crystallites of the particles [35]. Actually, we used several mg particles for XRD characterization. With the same amount, a well featured XRD spectrum of Ag particles synthesized by LAL of Ag in acetone has been observed [36], which indicates that the particles synthesized by LAL of Fe in acetone are in very low crystallinity.

**Figure 2:**
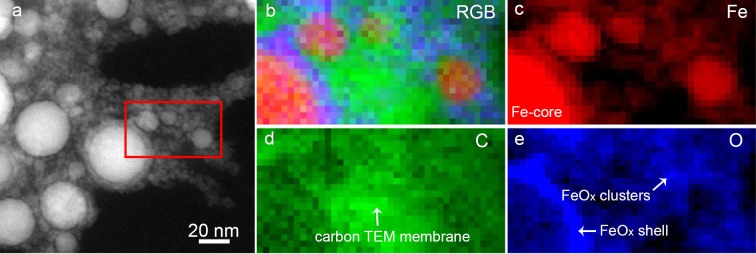
(**a**) TEM image and (**b**–**e**) EDX mapping of small clusters. (**b**) TEM image of mixed elements of (**c**) Fe, (**d**) C and (**e**) O images.

**Figure 3:**
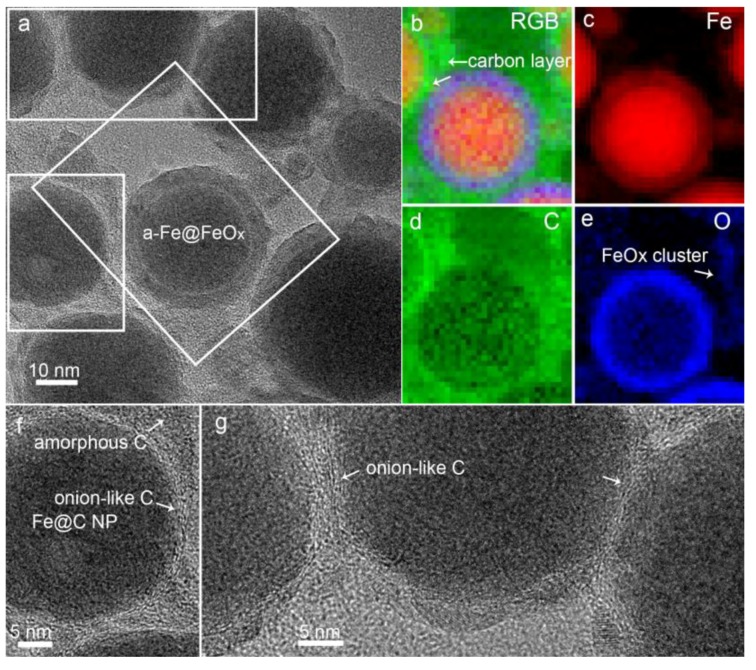
(**a**) TEM image and (**b**–**e**) EDX mapping of a Fe@FeO_x_ core-shell particle. (**b**) TEM image of mixed elements of (**c**) Fe, (**d**) C and (**e**) O images. (**f**,**g**) TEM images of the Fe@C particles.

To confirm the compositions of small clusters and core-shell particles, the distribution of Fe, C, O elements in the nanoblends was analyzed by EDX as shown in Figure 2 and Figure 3. As indicated by TEM image (Figure 2a) and the Fe and O elemental distributions (Figure 2c,e), the small clusters were identified as FeO_x_. Besides this, a certain amount of carbon was also detected (Figure 2b,d). However, given that the particles were deposited on the carbon membrane of a TEM grid, it is difficult to differentiate whether the detected carbon comes from the particles or not.

Apparent evidence for the generation of carbon during LAL was witnessed by HRTEM characterization (Figure 3a,f,g) and EDX analysis (Figure 3b–e). Both amorphous carbon (Figure 3b,d,f) and onion-like carbon (Figure 3b,d,f,g) were discovered, which encapsulated amorphous Fe particles to form the amorphous-Fe@carbon (a-Fe@C) core-shell particles (a representative particle is shown with an arrow marked in Figure 3b). Facilitated by the adhesion of different carbon shells, a-Fe@C core-shell particles gradually evolve into a particle network (Figure 1a–c and Figure 3g). This phenomenon is consistent with previous reports that LAL in organic solvents often causes the decomposition of solvent molecules and results in the formation of carbon-encapsulated particle networks [10,37]. Amorphous FeO_x_ clusters (Figure 3a–e) were also generated, which surrounded big core-shell particles to facilitate the formation of particle network. Figure 2a–e and Figure 3a–e also indicate that, besides a-Fe@C core-shell particles, large Fe@FeO_x_ core-shell particles with diameters of tens of nm are produced by LAL of Fe in acetone.

**Figure 4:**
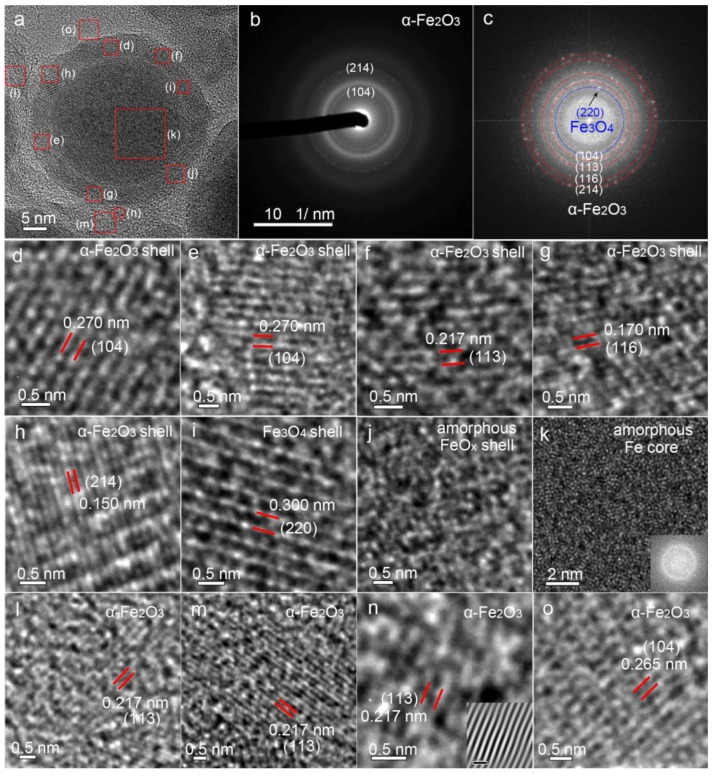
(**a**) High-resolution transition electron microscropy (HRTEM) image of a Fe@FeO_x_ core-shell particle, (**b**) selected area electron diffraction (SAED) and (**c**) FFT analysis of the core-shell particle. HRTEM images of (**d**–**j**) α-Fe_2_O_3_ shells, (**i**) Fe_3_O_4_ shell and (**j**) amorphous FeO_x_ shell and (**k**) amorphous Fe core, respectively. (**l**–**o**) Single-crystalline α-Fe_2_O_3_ crystals. Inset images in (**k**,**n**) show the FFT image indicating an amorphous structure and the inverse FFT image of the structure showing clearer crystal planes, respectively. The scale bar in the inset image of (**n**) is 0.5 nm.

To better understand the compositions of Fe@FeO_x_ core-shell particles, a core-shell particle was selected as the representative particle for HRTEM, SAED and FFT characterizations, as shown in Figure 4a–c. To further clarify the crystallinity in different regions, HRTEM images of seven domains of FeO_x_ shells and one larger domain of an Fe core were displayed in Figure 4d–k. The SAED pattern of the core-shell particle indicates that the core-shell particle has a low crystallinity since only two diffraction rings were observed (Figure 4b), which fits well with the (104) and (214) planes of α-Fe_2_O_3_ (ICSD No. 01-089-0597). FFT analysis gives more information about the crystallinity of the core-shell particle, which indicates that two more planes of the (113) and (116) planes of α-Fe_2_O_3_ are also present. Figure 4d–h display the HRTEM images of different crystal domains in the FeO_x_ shell which possess (104), (113), (116) and (214) planes of α-Fe_2_O_3_ with interplanar distances of 0.270, 0.217, 0.170 and 0.150 nm, respectively. Besides α-Fe_2_O_3_, another diffraction ring belonging to a crystal plane with an interplanar distance of 0.300 nm was also detected, which can be assigned to the (220) plane of Fe_3_O_4_ (ICSD No. 01-089-0950). It is noteworthy that (i) the crystals in the FeO_x_ shell are mainly single crystalline; and (ii) the quality of the single-crystallinity is not particularly good because many defects among the crystal planes are obvious (Figure 4d–g,l–o). One domain of the FeO_x_ shell is almost completely amorphous (Figure 4j). As concluded from Figure 4d–h, the FeO_x_ shell is composed of many α-Fe_2_O_3_ single-crystalline crystals with different crystallographic orientations. Regarding the Fe core, it is totally amorphous (Figure 4k). That is why no peak was detected during XRD characterization (Figure 1e). Regarding the dominant small particles, four crystalline domains outside the Fe@FeO_x_ particle are shown in Figure 4l–o. The interplanar distances of 0.217, 0.217, 0.217 and 0.270 nm of these crystalline domains are well indexed to (113), (113), (113) and (104) planes of α-Fe_2_O_3_. As indicated by HRTEM analysis, it is clear that single-crystalline α-Fe_2_O_3_ nanocrystals with different crystallographic orientations are abundant in the nanoblends.

**Figure 5:**
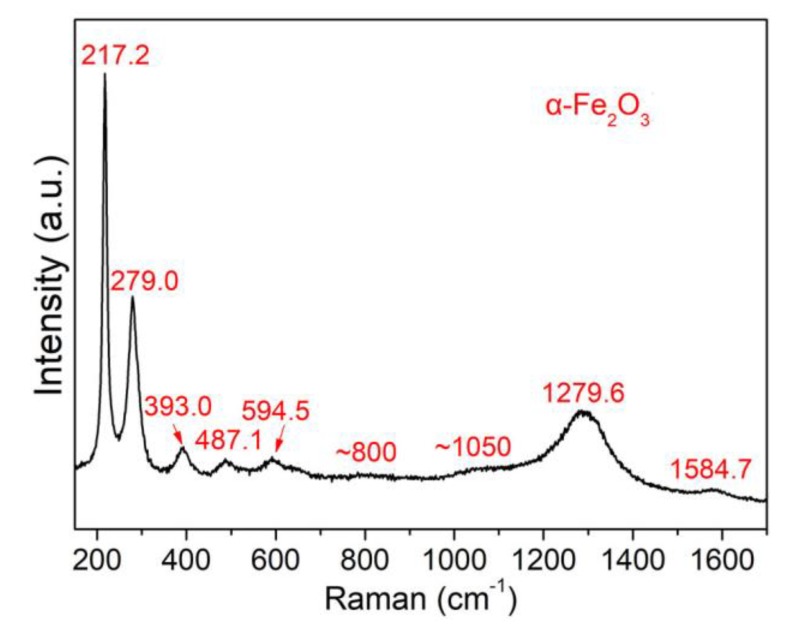
Raman spectrum of the particles obtained by the laser ablation in liquids (LAL) of Fe in acetone.

**Figure 6:**
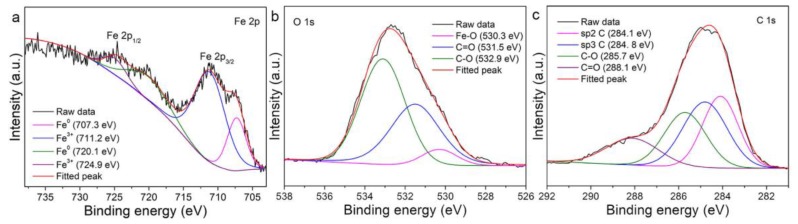
High resolution XPS (**a**) Fe 2p, (**b**) O 1s and (**c**) C 1s spectra from the colloids synthesized by laser ablation of Fe in acetone.

Raman spectroscopy shows seven peaks at 220.9, 239.5, 286.0, 401.4, 495.9, 606.7, 659.4, 812.0, 1050, 1099.4, 1297.7 and 1603.0 cm^−1^ (Figure 5) associated with α-Fe_2_O_3_, in accordance with the conclusion from HRTEM analysis (Figure 4) that α-Fe_2_O_3_ is the main crystalline product. Besides the peaks corresponding to α-Fe_2_O_3_, another small peak is also observed at 1584.7 cm^−1^, which can be assigned to the G-band of carbon and therefore indicates the presence of a large amount of carbon in the particles, in accordance with the HRTEM images shown in Figure 3f–g and XPS analysis which show that the outermost 5 nm-thick surfaces of the as-prepared particles are composed of 72.22% C, 1.29% Fe and 26.49% O. The high C ratio and low Fe and O ratios suggest that a higher ratio of carbon/carbon-byproduct clusters are generated by the laser-induced decomposition of acetone molecues. High-resolution XPS Fe 2p, O 1s and C 1s spectra are shown in Figure 6. After peak fitting, the ratio of sp^2^/sp^3^-C was calculated to be 0.89. The sp^2^ and sp^3^ carbons correspond to an ordered graphite (sp^2^) structure and disordered graphite layers (e.g., soot, chars, glassy carbon, and evaporated amorphous carbon [36,38]), respectively. Thus, more than half of the C that precipitates on Fe@Fe_2_O_3_ particles is crystalline because the sp^2^/sp^3^ ratio is less than 1, in accordance with the HRTEM images shown in Figure 3f–g, where onion-like carbons with some defects appear as shells to embed a-Fe particles inside. When both sp^2^ and sp^3^ C states are mixed in particles, diamond-like carbon (DLC) [39] structures are considered to be generated. DLC structures should be a typical product of LAL in organic solvents since they were also observed from other nanomaterials obtained by the LAL of different metals (e.g., Ag [36], Mo [39], Ti, [40], Ta [41], Nb [41], Hf [41], Mo [41] and Co [37]) in organic solvents. Peak fitting of C 1s (Figure 6c) shows that 15.91% and 21.90% of the carbon have C=O and C–O bonding, respectively, which is due to adsorbed acetone molecules and their decomposition byproducts. From the XPS Fe 2p spectrum (Figure 6a), only the binding energies that correspond to Fe^3+^ (711.2 eV and 724.9 eV) and Fe^0^ (707.3 eV and 720.1 eV) are observed, which come from α-Fe_2_O_3_ and a-Fe, respectively. Because of abundant C–O and C=O bindings on the particle surfaces, only a small amount of Fe-O binding can be deconvoluted from the O 1s spectrum (Figure 6b). Both XPS and Raman spectra support the conclusion from HRTEM analysis that α-Fe_2_O_3_ is the dominant phase of the crystalline particles.

**Figure 7:**
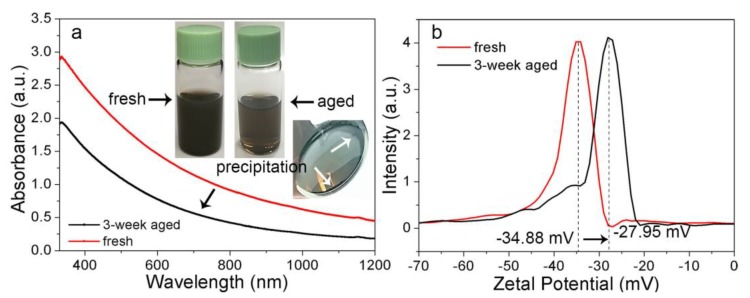
(**a**) Absorption spectra for the fresh colloid (red curve) synthesized by laser ablation of Fe in acetone at 600 mW and (**b**) the colloid stored for 3 weeks (black curve), respectively. Inset images in (**a**) are optical images of the fresh colloid (left) and the colloid stored for 3 weeks (middle), where the precipitation of the colloid (as indicated by white arrows in the right optical image) causes the downshift of the absorbance spectra. (**b**) Zeta potential curves of the fresh colloid and the colloid stored for 3 weeks.

The synthesized Fe@α-Fe_2_O_3_ and α-Fe_2_O_3_ particles were unstable with gradual particle precipitation at the bottom of the glass container (right optical image inset in Figure 7a) during the colloid storage. As a result, the absorbance spectrum of the colloid downshifted (Figure 7a) and the optical transparency of the colloid increased after 3-week storage (left and middle optical images inset in Figure 7a). Additionally, the zeta potential values of the colloid decreased from −34.88 mV to −27.95 mV after 3-week storage, which indicates the decreased stability of the colloid. The zeta potential is indicative of the difference in the electric potentials between the charges of the species which strongly adsorb on the particle surface and those (with the opposite sign) of the diffuse layer in the dispersing medium [42]. It is often considered that the colloids with a zeta potential value smaller than −30 mV or larger than 30 mV are stable, while those with zeta potential values in the range of −30~30 mV are unstable [3]. Hence, in principle, one would expect that the stable particles with larger charges remain well dispersed while those with lower charges precipitate. However, in our case, the zeta potential value of the colloids decreased over time, which indicated that the gradual aggregation of nanoparticles occurred during colloidal storage. The magnetic properties among magnetic particles and the “capture” behavior of both carbon shells (Figure 3a) and free carbon clusters [36] cause colloidal aggregation and precipitation during storage. Considering the excellent long-term (six-month) stability of Ag colloid produced by LAL in acetone [36], it is highly possible that the magnetostatic interaction among magnetic particles is the main reason to be responsible for the colloidal aggregation and precipitation.

Compared with the techniques of laser target evaporation in gases and laser ablation in air whereby metal-oxide [43,44] is produced, LAL is better at the synthesis of Fe@C and Fe@FeO_x_ core-shell particles and further indicates a new way to synthesize single-crystalline iron oxide particles.

### 3.2. Magnetic Properties

**Figure 8:**
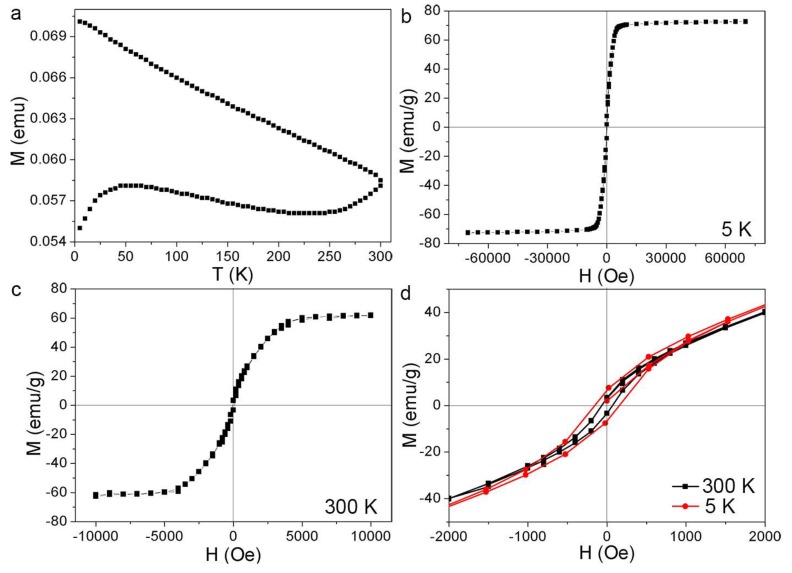
Magnetic characterization of the Fe@α-Fe_2_O_3_ particles synthesized by laser ablation in acetone. (**a**) ZFC/FC curves in 50 Oe, (**b**,**c**) hysteresis curves at 5 and 300 K. (**d**) Magnified hysteresis curves at 5 and 300 K.

The magnetic properties of the Fe@α-Fe_2_O_3_ particles prepared in acetone are presented in Figure 8. To reveal the relationship between magnetization and temperature, ZFC and FC curves of the as-prepared nanomaterials were measured. The ZFC curve in Figure 8a shows a broad maximum peaking at 52 K, followed by a plateau and a subsequent gradual increase from 250–300 K suggesting two different fractions of particles. The FC branch exhibits an almost linear increase of the magnetic moment with decreasing temperature from 300 K to 5 K. Figure 8b,c show the magnetic hysteresis loops at 5 K and 300 K with saturation magnetization M_S_ = 72.5 emu/g and 61.9 emu/g, respectively. The magnification around zero field delivers coercivities of H_C_ = 160 Oe at 5 K and 70 Oe at 300 K.

The structural and morphological studies above reveal a mixture of Fe@α-Fe_2_O_3_, Fe@C, and α-Fe_2_O_3_ particles (Figure 2, Figure 3 and Figure 4) which can be split into two size regimes. The sizes of large Fe@α-Fe_2_O_3_ and Fe@C core-shell particles with a number frequency of about 10% are in the range of 10–100 nm, with the maximum diameter of the distribution being 30 nm (Figure 1d). α-Fe_2_O_3_ particles are significantly smaller, with a medium size of 4–5 nm (Figure 1d), at a number frequency of about 90%. For the magnetometry of magnetic powders, magnetization is the ratio of the magnetic moment with respect to mass of particles in different size regimes. Taking identical mass densities as a rough estimate, the relative mass fraction of the large particles is calculated to be 87%–93%. Further consideration of the significantly smaller magnetization of α-Fe_2_O_3_ of 1 emu/g as compared to amorphous Fe with M_S_ > 100 emu/g [45] further reduces the relative magnetic signal from α-Fe_2_O_3_. This means that the vast majority of the magnetic signal stems from the larger particles while the smaller are expected contributing less than 1% in the saturated state.

Therefore, the saturation magnetization of M_S_ = 72.5 emu/g and 61.9 emu/g at 5 K and 300 K, respectively, is considered to mainly stem from the amorphous Fe cores of large a-Fe@FeO_x_ core-shell particles. This deduction is also helpful in understanding the magnetic properties (M_S_ = 67 emu/g at 300 K) of a-Fe@C and Fe_3_O_4_ nanoblends synthesized by fs-LAL of Fe in acetone under other conditions (35 fs, 800 nm, 5 kHz, 800 μJ/pulse) [18]. The remanence to saturation ratio (*M_r_*/*M_s_*), also called the saturation magnetization ratio, was calculated to be ca. 0.1, which was smaller than that (0.5) expected theoretically for randomly oriented single domain grains [46], which indicates the presence of a significant amount of superparamagnetic small particles, domain walls in large particles, and the occurrence of antiferromagnetic interactions [47]. Here, all the above may add to an overall low remanence. It is well known that α-Fe_2_O_3_ is weakly ferromagnetic or antiferromagnetic [48], and its presence as the shell material endows antiferromagnetic properties to the Fe@α-Fe_2_O_3_ particles while the small α-Fe_2_O_3_ particles are expected to be superparamagnetic at 300 K and thermally blocked at 5 K (see discussion below). The interfacial magnetic interactions between ferromagnetic cores and antiferromagnetic shells [49,50] may also endow high orbital magnetic moment to LAL-synthesized a-Fe@α-Fe_2_O_3_ particles. For the metallic a-Fe core, Grinstaff et al. have confirmed that glassy a-Fe is a soft ferromagnetic material with M_S_ = 152 emu/g and H_c_ = 160 Oe at T = 5 K [45]. While H_c_ fits well to the present results, the lower M_S_ can be explained by the mixture of amorphous Fe, the weakly ferromagnetic α-Fe_2_O_3_, and the unknown but significant amount of C in the sample. Thus, the remanent magnetization mainly originates from large Fe@α-Fe_2_O_3_ core-shell ferromagnetic particles with multi-domains, which cannot rapidly demagnetize by domain formation in the absence of an applied field.

A more interesting phenomenon is the magnetic signature of small α-Fe_2_O_3_ particles, which results in different ZFC/FC curves as compared to those of a-Fe@C particles synthesized by fs-LAL of Fe in acetone under other conditions [18]. The broad peak with a maximum at 52 K is ascribed to the blocking behavior of α-Fe_2_O_3_ particles on top of an almost constant signal in the interval of 5–250 K arising from the larger Fe@α-Fe_2_O_3_ particles. The smallest particles of the Fe@α-Fe_2_O_3_ particles gradually cross their blocking temperature T_B_ with the temperature increasing above 250 K. At 300 K, however, only a minor fraction of Fe@α-Fe_2_O_3_ is superparamagnetic, explaining the monotonous increase in the FC branch; moreover, the interparticle interactions [51] among LAL-generated particles [43] of the magnetic core-shell particles are supposedly not strong enough to cause collective magnetic freezing to enter a spin-glass state.

We do not observe any sharp change of the magnetization, which indicates the absence of the Morin transition in α-Fe_2_O_3_. Previous reports have shown that in small α-Fe_2_O_3_ particles with diameters below 19 nm (cf. Figure 1a–d), the Morin transition occurring in bulk α-Fe_2_O_3_ is smeared out over a wide temperature range or even completely suppressed [52,53]. This leads to weakly ferromagnetic α-Fe_2_O_3_ for all considered temperatures. The blocking behavior with T_B_ = 52 K at the peak position can be translated to an effective magnetic anisotropy energy density K_eff_ via K_eff_·V = 25 k_B_T_B_ with V the particle volume and k_B_ Boltzmann’s constant. The factor of 25 is the natural logarithm of the product of the measurement time window of SQUID magnetometry (10 s) and the intrinsic attempt frequency of about 10^10^ Hz [53]. For 4–5 nm hematite nanospheres, as in our cases, K_eff_ is calculated to be 2.7–5.4 × 10^5^ J/m^3^. Bödker et al. extracted an energy barrier of 300–600 K for 16 nm which translates to K_eff_ = 0.5–1.1 × 10^5^ J/m^3^ [53] and a strongly increasing K_eff_ when the particle size is reduced, reaching a maximum value of 2.4 × 10^5^ J/m^3^ for 5.9 nm particles at the smallest investigated diameter [54]. In this light, the obtained results for the 4–5 nm α-Fe_2_O_3_ particles convincingly extend the size dependence to smaller diameters.

Despite a very broad size distribution, the α-Fe_2_O_3_ particles synthesized by fs-LAL possess the lowest blocking temperature of 52 K among all α-Fe_2_O_3_ particles synthesized by LAL [2]. The blocking temperature of FeO_x_ particles synthesized by the ns-LAL of Fe in water was ca. 220 K, which corresponds to the particle size of 15 nm [14]. The ps-LAL of Fe in ethanol gave rise to the formation of Fe_3_O_4_/Fe_3_C mixture colloids which had a bimodal size distribution with maxima at ca. 3 nm and ca. 12 nm [21]. Because of the generation of a greater amount of small particles by ps-LAL, the blocking temperature down-shifted to 120 K when the applied field was 50 Oe [21]. According to the previously reported relationship between the blocking temperatures and particle sizes [20], it is estimated that the average size of Fe_3_O_4_/Fe_3_C mixture colloid obtained by ps-LAL in ethanol was ca. 9 nm. In the case of fs-LAL shown in this work, the blocking temperature is further lowered to 52 K, which corresponds to the particle size of ca. 4–5 nm for α-Fe_2_O_3_.

### 3.3. Formation Mechanism

Considering the advantage of fs-LAL over ps-LAL, and ns-LAL enabling the synthesis of ultrasmall magnetic particles with lower blocking temperatures, the formation mechanism of both Fe@C, Fe@FeO_x_ and ultrasmall α-Fe_2_O_3_ particles is here proposed to show the uniqueness of the fs-LAL process. The large size difference between ultrasmall α-Fe_2_O_3_ clusters of several nm and large core-shell particles with sizes ranging from tens of nm to 130 nm indicates that large particles do not form through particle growth mechanism but form through ejection of large Fe particles during LAL [55]. The ultrasmall α-Fe_2_O_3_ particles less than 10 nm (Figure 5g) should form due to phase/Coloumb explosion mechanism for fs-LAL. In contrast, owing to the thermal ablation mechanism, the sizes of the majority of the small particles inside the cavitation bubble are already 12 nm for ns-LAL [56]. The particle growth after bubble collapse often leads to a further increase in the particle sizes. Therefore, despite a small amount of large Fe particle ejection due to thermal effects during fs-LAL, the main “cold” process of fs-LAL is more efficient at generating ultrasmall particles than both ps- and ns-LAL.

**Figure 9:**
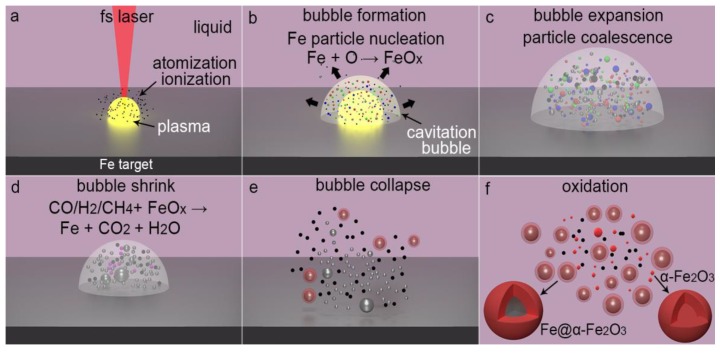
(**a**–**f**) Schematic of formation mechanism for Fe@α-Fe_2_O_3_ particles by fs-LAL of Fe in acetone. Oxygen radicals that react with Fe atoms to from FeO_x_ come from fs laser induced decomposition of acetone and dissolved oxygen. Fe: grey color, C: black color, α-Fe_2_O_3_: red color, other FeO_x_ phases: green and blue color. Note that the shockwaves [59] generated during bubble collapse can push the already formed α-Fe_2_O_3_ ultrasmall particles towards Fe@FeO_x_ particles to be captured by the FeO_x_ shells.

Due to the plasma-induced decomposition of acetone molecules and the dissociation of the dissolved oxygen (Figure 9a) [57], O radicals are generated during fs-LAL of Fe in acetone, which may react with the surrounding Fe atoms (generated from plasma-induced target material atomization) to form FeO_x_ clusters (Figure 9b). However, due to the existence of limited oxygen, the main products generated from the plasma phase are pure Fe clusters. The sizes of Fe and FeO_x_ clusters may increase slightly during bubble expansion (Figure 9c) by coalescence. Inside the cavitation bubble, reductive gases [58] such as H_2_, CO and CH_4_ reduce FeO_x_, which results in the formation of pure Fe particles (Figure 8d). After bubble collapses (Figure 8e), the outer parts of large Fe particles are oxidized into FeO_x_ shells containing a large amount of α-Fe_2_O_3_ domains (Figure 9g), while the ultrasmall Fe particles are oxidized into α-Fe_2_O_3_ particles (Figure 9f,g). Due to the difference in local temperature and pressure as well as the variation in oxygen abundance around Fe particles, Fe particles crystallize into α-Fe_2_O_3_ or Fe_3_O_4_ single crystals along different crystallographic orientations. It is also possible that (1) reductive gases in the cavitation bubbles inhibit complete oxidation of Fe into FeO_x_ and their further polycrystallization; (2) during bubble collapses, the shock waves [59] render small α-Fe_2_O_3_ crystals with high kinetic energy to make them quickly eject towards the already formed Fe@FeO_x_ particles to be captured by FeO_x_ shells as single domains.

Extremely superfast cooling of the molten Fe droplets inhibits their crystallization so that a-Fe rather than crystalline Fe particles form after LAL. The cooling rate required for a-Fe formation ranges from 10^5^ to 10^7^ K/s [60], which can be easily obtained during the ultrafast quenching of LAL-generated plasma (thousands of Kelvin quenching within a submicro-second interval [61]). During the ejection of the molten Fe particles from the ablated target, they interact with acetone molecules, as a case of the electric explosion of steel in carbon-rich liquids [62,63], resulting in the formation of carbon atoms and other carbonaceous byproducts, which then precipitate on Fe particles to form C-shells. During their precipitation, Fe particles with high surface activity act as catalysts to facilitate the formation of onion-like carbon shells. The presence of carbons on the Fe particles inhibits particle growth and coalescence [4] and prevent surface oxidation, leading to the formation of Fe@C core-shell particles (Figure 3f–g).

Under the impact of the high-temperature and high-pressure environment in the plasma phase of fs-LAL, the atomization/ionization of carbon impurities in the iron substrates and the decomposition of the acetone molecules [64,65] occur simultaneously (Figure 9a), which leads to the formation of free C clusters. Besides pure carbon, polycyclic structures may be also generated through the interaction between Fe particles and organic solvents [63], which may precipitate on the already particles to make them evolve into networks. In consequence, the stability of the colloids decreases during storage and results in the precipitation of particles (Figure 7).

## 4. Conclusions

This work has demonstrated the capability of synthesizing large Fe@α-Fe_2_O_3_ and small α-Fe_2_O_3_ particles which are practically completely split in the size histogram. Four to five nanometer α-Fe_2_O_3_ particles exhibit a low blocking temperature of 52 K by fs-LAL in acetone, among the lowest ever achieved by LAL. From superparamagnetic blocking, an effective magnetic anisotropy K_eff_ = 2.7–5.4 × 10^5^ J/m^3^ has been estimated which extends previous investigations convincingly towards smaller hematite particle sizes. Surprisingly, most small α-Fe_2_O_3_ particles were single-crystalline, and so the possibility of synthesizing single-crystalline particles by LAL was demonstrated. Because of the dominant mass of large Fe@α-Fe_2_O_3_ and Fe@C particles (10–100 nm), all nanoblends show a soft magnetic behavior with saturation magnetization (M_s_) and coercivities (H_c_) values of 72.5 emu/g and 160 Oe at 5 K and 61.9 emu/g and 70 Oe at 300 K, respectively, which mainly originate from amorphous Fe core particles. Previously, ZFC/FC curves were seldom investigated as compared to hysteresis curves for LAL-generated magnetic particles. Here, it was shown that the blocking temperatures in the ZFC curves can be used to estimate the sizes of small magnetic particles.
